# An Army Medical Reserve Corps

**Published:** 1904-04

**Authors:** 


**A Monthly Review of Medicine and Surgery.**

*EDITOR:*

WILLIAM WARREN POTTER, M. D.

All communications, whether of a literary or business nature, books for review and exchanges should be addressed to the editor: 284 FRANKLIN STREET, BUFFALO, N. Y.

VOL. XLIII.—LIX. APRIL, 1904. No. 9.

### An Army Medical Reserve Corps.

AT LAST, and in all probability in response to the very general demand for reform on the part of the medical press of this country, a bill has been prepared and introduced in congress, providing for the recognition of the civilian surgeon in regular army life. The agitation for reform in this direction was begun during the Spanish war, when the necessity for additional medical officers required the employment of a large number of physicians from civil life who were known as acting assistant surgeons, with the relative rank and pay of a first lieutenant and assistant surgeon in the medical department.

These men were by special order allowed to wear the uniform of the United States army with the exception that their shoulder straps and collar ornaments were of silver instead of gold, as worn by the regularly commissioned surgeon. They were allowed quarters, but nothing else; they were not entitled to pension in the event of injury on the battle field; they were considered as contract employes, and, in fact, the term contract surgeon was more often applied to them in unofficial designation than that of acting assistant surgeon, as they were termed in official correspondence. They were merely attached and had no standing whatever, except that given them by Surgeon-General Sternberg, whose request to the Adjutant General that an order be issued requiring obedience to the orders of the acting assistant surgeon, was complied with and his position as a semi-attached officer was established. The enlisted man understood thoroughly the position of the civilian surgeon and while he recognised his worth and his ability, yet he was not a commissioned officer, and that in the eyes of the soldier made a great difference in his standing, officially and socially, even to a large number of commanding officers.

After the Spanish War, when surgeons were needed in the Philippines, the designation was changed from acting assistant surgeon to that of contract surgeon. This added indignity was placed upon the civilian surgeon with a cold-blooded deliberateness, which was chilling to say the least. They were stripped of their imitation uniform; they were not allowed any of the rights and privileges the acting assistant surgeon was supposed to have enjoyed. They were made to feel that they were simply temporary conveniences, and the climax was reached when the second auditor of the treasury, an office which has for years been considered by the army man as the headquarters of official assininity, issued a decision that the contract surgeon was merely a civilian employe under contract, the same as a teamster, for instance, and as such enlisted men and nurses and hospital corps men need not obey his orders. To the credit of the men of the hospital corps and the stewards under whose direction they work, there has not been reported a single instance where the orders of a contract surgeon have been disobeyed in spite of this interpretation from the treasury department.

These successive moves, made in opposition to the wishes of the surgeon general it is understood, nearly ruined the chances of the United States Army of securing competent medical officers in case of necessity. Certainly no self-respecting surgeon would take service under such circumstances. The bill which has been introduced in congress and which is urged by Surgeon-General O'Reilly, places the civilian surgeon on an equal footing with the regular army surgeon during the time he is in service. It provides for the formation of a medical reserve corps the members of which shall be commissioned as first lieutenants and assistant surgeons; they will have all the privileges of the regular army officer, except that of promotion. They have been accorded even the increase of pay which goes with long service. Contract surgeons now in the service and acting assistant surgeons of the Spanish war who made good records may be commissioned on recommendation of the surgeon general; all others who enter will have to pass an examination as to fitness. No officer of the reserve corps will be entitled to retirement or retirement pay, but he will be entitled to pension when physical disability is incurred while on active service and in line of duty. The bill is one of the most important which has been presented in connection with the medical department of the army in years, and will insure for the service prompt and competent medical equipment in case of war.

A commission in the proposed reserve corps will give the officer the privileges of the Army Medical School at Washington, and the surgeon general of the army will probably arrange for the instruction of the new officers in the complicated paper and executive work of the service, lack of knowledge of which on the part of the acting assistant surgeon who was so unfortunate as not to have the advice of a seasoned hospital steward, resulted in great property loss to the government, and an almost endless muddling of official papers and reports.

